# A GENETICALLY INFORMED STUDY ON THE ETIOLOGICAL ASSOCIATIONS AMONG EPIGENETIC CLOCKS

**DOI:** 10.1093/geroni/igad104.2478

**Published:** 2023-12-21

**Authors:** Eric Penichet, Christopher Beam, Kelly Bakulski, Eric Turkheimer, Kristin Higdon, Lesa Ryan, Kendra Sikes, Deborah Davis

**Affiliations:** University of Southern California, Los Angeles, California, United States; University of Southern California, Los Angeles, California, United States; University of Michigan, Ann Arbor, Michigan, United States; University of Virginia, Charlottesville, Virginia, United States; Norton Children’s Research Institute, Louisville, Kentucky, United States; University of Louisville School of Medicine, Louisville, Kentucky, United States; Norton Healthcare, Louisville, Kentucky, United States; University of Louisville School of Medicine, Louisville, Kentucky, United States

## Abstract

Epigenetic clocks are believed to capture the molecular footprints of biological programs that subserve innate aging processes. Although epigenetic clocks can predict disease phenotypes and mortality with a high degree of accuracy, little is known about the extent to which they capture the same (or different) developmental and maintenance mechanisms. Quantitative genetic studies may provide useful information on the etiological links between epigenetic clocks, particularly pertaining to the question of whether they are influenced by a common set of genetic and environmental factors. Using preliminary data from the Louisville Twin Study, this study used a multivariate independent pathway model to examine the shared and unique genetic and environmental influences of three epigenetic clocks (GrimAge, HannumAge, PhenoAge). Participants consisted of 43 monozygotic pairs and 30 (18 same-sex) dizygotic twin pairs with a mean age of 54.5 years (sd = 5.27). Phenotypic analyses revealed significant correlations among the clocks (r = 0.63 - 0.78). Subsequent multivariate biometric analyses showed that there were common genetic and nonshared environmental influences across all three clocks (p < 0.001), with unique genetic and nonshared environmental influences specific to GrimAge and HannumAge (p < 0.001). To examine the sources of covariation between these variables, biometric regression models were used to decompose the covariance between each of the clocks (GrimAge and Hannum, Hannum and PhenoAge, PhenoAge and GrimAge) into genetic and environmental components. These analyses revealed large, significant genetic correlations (rg = 0.80-0.86) and medium to large, significant nonshared environmental correlations (re = 0.37-0.73) among the clocks.

